# Vitamin D status is inversely associated with markers of risk for type 2 diabetes: A population based study in Victoria, Australia

**DOI:** 10.1371/journal.pone.0178825

**Published:** 2017-06-02

**Authors:** Poonam K. Pannu, Leonard S. Piers, Mario J. Soares, Yun Zhao, Zahid Ansari

**Affiliations:** 1 Food, Nutrition & Health, School of Public Health, Curtin Health Innovation Research Institute, Curtin University, Perth, Western Australia, Australia; 2 Health Intelligence Unit, System Intelligence and Analytics Branch, Health Strategy, Productivity and Analytics Division, Department of Health, Melbourne, Victoria, Australia; 3 Occupation and the Environment, School of Public Health, Faculty of Health Sciences, Curtin University, Perth, Western Australia, Australia; University Sains Malaysia, MALAYSIA

## Abstract

A growing body of evidence suggests a protective role of vitamin D on the risk of type 2 diabetes mellitus (T2DM). We investigated this relationship in a population sample from one Australian state. The data of 3,393 Australian adults aged 18–75 years who participated in the 2009–2010 Victorian Health Monitor survey was analyzed. Socio-demographic information, biomedical variables, and dietary intakes were collected and fasting blood samples were analyzed for 25, hydroxycholecalciferol (25OHD), HbA1c, fasting plasma glucose (FPG), and lipid profiles. Logistic regression analyses were used to evaluate the association between tertiles of serum 25OHD and categories of FPG (<5.6 mmol/L vs. 5.6–6.9 mmol/L), and HbA1c (<5.7% vs. 5.7–6.4%). After adjusting for social, dietary, biomedical and metabolic syndrome (MetS) components (waist circumference, HDL cholesterol, triglycerides, and blood pressure), every 10 nmol/L increment in serum 25OHD significantly reduced the adjusted odds ratio (AOR) of a higher FPG [AOR 0.91, (0.86, 0.97); p = 0.002] and a higher HbA1c [AOR 0.94, (0.90, 0.98); p = 0.009]. Analysis by tertiles of 25OHD indicated that after adjustment for socio-demographic and dietary variables, those with high 25OHD (65–204 nmol/L) had reduced odds of a higher FPG [AOR 0.60, (0.43, 0.83); p = 0.008] as well as higher HbA1c [AOR 0.67, (0.53, 0.85); p = 0.005] compared to the lowest 25OHD (10–44 nmol/L) tertile. On final adjustment for other components of MetS, those in the highest tertile of 25OHD had significantly reduced odds of higher FPG [AOR 0.61, (0.44, 0.84); p = 0.011] and of higher HbA1c [AOR 0.74, (0.58, 0.93); p = 0.041] vs. low 25OHD tertile. Overall, the data support a direct, protective effect of higher 25OHD on FPG and HbA1c; two criteria for assessment of risk of T2DM.

## Introduction

Vitamin D status, as judged from circulating concentrations of 25, hydroxycholecalciferol (25OHD), is a worldwide concern. In many countries across all continents, approximately 50% of those populations have an inadequate 25OHD status (<50 nmol/L) [1]. Countries like India and China have some of the highest rates of vitamin D deficiency (<25 nmol/L) [1]. The 25OHD status of Australians is also surprisingly low for a country blessed with abundant sunshine. Current estimates indicate that ~31% of the population have inadequate 25OHD levels [2], with a higher prevalence in older Australians [3]. The prevalence of type 2 diabetes mellitus (T2DM) has also risen tremendously in the last 10 years, with projections that countries like India and China will have the highest numbers by 2030 at 79.4 and 42.3 million respectively [4].

There is an ongoing interest in the extra-skeletal effects of vitamin D including its potential to blunt the risk of developing T2DM. Positive outcomes would present a tangible public health solution, if causality is accepted. Such a relationship was first suggested in 1967 by Milner and Hales, who found that insulin secretion in rabbits was dependent on calcium and magnesium, which are tightly regulated by vitamin D [5]. Accumulating evidence has indicated that higher 25OHD status may have several anti-diabetic effects, including improvement in insulin sensitivity, stabilizing HbA1c levels [6], and improving beta cell function [7], whereas low 25OHD status may increase risk of T2DM [8]. Thus, in the current environment of increasing rates of T2DM [9], their close parallelism with insufficient levels of 25OHD deserves investigation in population based studies. There are several lifestyle factors that modulate the risk of T2DM, including dietary components and patterns [10, 11], physical activity, and smoking [12]. The risk of developing T2DM over 20 years appears to increase with the accumulation of metabolic syndrome (MetS) components. The risk of T2DM increased by: 11.9% in those with zero Mets components, 31.2% in those with three MetS components and 40.8% in those with four or five MetS components [13]. Though the presence of MetS components increases the risk of T2DM, glucose is the most strongly correlated factor in predicting the development of diabetes in the future [14]. In a study of more than 58,000 adults, as the number of components increased, so did the incidence of diabetes [14]. However, some gaps may exist with one study in Hispanic Americans finding that impaired glucose tolerance had a greater predictive power than the individual MetS components [15]. Thus, the presence of MetS is another major risk predictor of increased T2DM [16].

The aim of this study was to investigate the association of 25OHD, and the risk of T2DM. We [17] as well as others [18–21], have shown that higher 25OHD status significantly reduced the risk of MetS and its components. Hence it was essential to adjust for several lifestyle factors and components of MetS, other than glucose, in order to correctly identify any independent association between 25OHD and risk of T2DM. One other study [8] has also investigated the association between 25OHD and T2DM and adjusted for three out of the four MetS components. Thus our study appears to be one of the first to adjust for all MetS components. Impaired fasting plasma glucose (FPG) and HbA1c levels are now recommended as key determinants of early risk of T2DM [22]. While high FPG is an immediate indicator of poor glucose homeostasis, HbA1c is a better indicator of longer term control of blood glucose, and recommended cut-offs for both these biomarkers are used to diagnose T2DM [22]. The underlying hypothesis of the present investigation was that increases in 25OHD would reduce the odds ratio of a high FPG and a high HbA1c after adjustment for socio-demographic, dietary and biomedical confounders.

## Materials and methods

### Sample

The Victorian Health Monitor (VHM) survey was a state-wide cross-sectional population based study [23] conducted in Victoria, Australia. Victoria lies in the south-east of Australia, and has a latitude of 37°47'S and longitude of 144°58'E. Data was collected between May 2009 and April 2010 including: physical information, dietary behaviour information and biomedical information. The physical and biomedical information of participants were collected by trained staff at four training sites. The VHM employed a stratified cluster sample selection method of Census Collection Districts within eight Department of Health regions in Victoria. Data were collected on 3,653 adults aged 18–75 years. From this sample, we excluded participants with: missing HbA1c and FPG data (n = 31), those with HbA1c ≥6.5% (n = 39) and FPG >7 mmol/L (n = 16) as they were classified as having T2DM as per the American Diabetes Association (ADA) cut-offs [22], those with T2DM (n = 140), those with type 1 diabetes (n = 9), and those on diabetic medications (n = 25). A total of 3,393 subjects were included in this analysis. Further details on physical, dietary, and biomedical data collection and analysis have been previously described [17, 23, 24].

### Biomedical measurements

Participants attended a testing site after an overnight fast of at least 10 hours. Blood samples were collected by venepuncture, and were subsequently transported to a central laboratory in Melbourne, Australia. Bloods were analysed for: FPG, HbA1c, 25OHD, high density lipoprotein cholesterol (HDL-C), and triglycerides (TG). The components in the blood were measured as follows: FPG using the hexokinase method, HbA1c using immunosassay (Roche Integra chemistry analyser), 25OHD concentration were measured based on the DiaSorin Corporation Liaison^®^ 25OHD total assay HDL-C using elimination/catalase method; and TG using GPO Trinder reagent set with serum blank. The blood pressure (BP) of participants was measured by survey staff using an automated BP monitor, which was calibrated regularly [23].

### Physical measurements

The anthropometric measurements were made at the testing sites by trained staff, and included height, weight and waist circumference. Height was measured without shoes using a stadiometer. Weight was measured without shoes and light clothing, using a digital weighting scale. Waist circumference was measured using a steel measuring tape. Body mass index (BMI) was calculated from the weight and height measurements [23].

### Dietary and physical activity measurements

Dietary information was collected by multiple-pass 24 h diet recall using computer assisted telephone interviews. Dietary recall interviews were conducted by dietitians from the Department of Nutrition and Dietetics at Monash University in Melbourne, Australia. The FoodWorks^®^ nutrition software (FoodWorks^®^ Interview) were used for conducting the dietary recalls. Based on the three dietary recalls, the mean intake for each nutrient was calculated and used in the analysis [23]. Further information on the assessment of dietary intake data can be found in our previous publications [17, 23]. Physical activity information was collected via interviews with the participant. The time spent in physical activity was calculated based on the sum of the time spent walking or performing moderate activity plus double the time spent in vigorous activity (to indicate its greater intensity) [23, 25].

### FPG and HbA1c

Fasting plasma glucose and HbA1c were the two dependent variables. The ADA cut-offs were used to identify those who were at a risk of T2DM based on FPG and HbA1c levels[22]. A binary variable was used to categorize subjects as being at low or high risk for T2DM: FPG <5.6 mmo/L (low risk, normal), vs. 5.6–6.9 mmol/L (high risk), and HbA1c <5.7% (low risk, normal) vs. 5.7–6.4% (high risk).

### 25OHD concentration

25OHD concentration was the primary independent variable. 25OHD concentration were categorized as tertiles: low 25OHD (median 33 nmol/L; range 10–44 nmol/l), medium 25OHD (median 54 nmol/L; range 45–65 nmol/L) and high 25OHD (median 77 nmol/L; range 65–204 nmol/L).

### Statistical analysis

#### Socio-demographic factors

In our analysis we considered a number of confounders, based on our [26] and others experience in the area [12, 27]. We adjusted for the following socio-demographic factors: age, gender, county of birth, Index of Relative Socio-economic Disadvantage (IRSED), physical activity, smoking status, and season. Age were entered as continuous variables. Country of birth was categorized as those born in Australia or overseas. The socio-economic indicator used was the IRSED, which is an index based on the social and economic conditions of individuals within an area [28]. Subjects were categorized into IRSED quartiles: quartile 1 (most disadvantaged), quartile 2 (disadvantaged), quartile 3 (less disadvantaged), and quartile 4 (least disadvantaged). Physical activity levels were classified into three categories: sufficient activity (≥150 minutes/week), insufficient activity (1–149 minutes/week), and inactive (0 minutes/week). There is a known seasonal variation to FPG and HbA1c, so we had to adjust for season [29, 30]. Season of biomedical assessment refers to the season of the year that the participant attended the testing site, and had their bloods collected for assessment of 25OHD status. Season of biomedical assessment were grouped as summer, autumn, winter, and spring. Smoking status were categorized into three categories: current smoker, ex-smoker, and non-smoker.

#### Dietary factors

Based on previous research [31–33] dietary factors included in the analyses were: dietary fiber, magnesium, alcohol, calcium, zinc, carbohydrate intake, energy intake and under/over reporting of energy intake. Self-reported energy intake may result in the under or over reporting of true energy intake (EI), and this may confound the estimation of any diet and disease related outcomes [34]. We predicted basal metabolic rate (BMR) using the Henry/Oxford equations based on a range of ages (18–30, 30–60, 60–70, and 70 and above years), gender and body weight [35]. Rather than use the Goldberg cut-offs to identify under-reporters and over-reporters [36], we calculated the ratio of energy intake to BMR (EI:BMR) and treated it as a confounder. Dietary fiber, magnesium, alcohol, calcium, zinc, carbohydrate intake, energy intake, and under/over reporting of energy intake were all entered as continuous variables.

#### Biomedical factors

Biomedical factors included in the analyses were: MetS components including waist circumference, high density lipoprotein (HDL) cholesterol, triglycerides (TG), BP, as well as body mass index (BMI), and haemoglobin levels (HbA1c model only). Haemoglobin levels were adjusted for in the HbA1c model only, as recent findings have indicated that haemoglobin levels may increase HbA1c levels [37, 38]. BMI and haemoglobin levels were entered as a continuous variable. Those with MetS tend to be at higher risk of developing T2DM, thus we adjusted for MetS components in the HbA1c and FPG model [39]. MetS components were each classified as binary variables, as defined by the joint interim statement [40]. Waist circumference were: normal waist circumference (<94cm for males or if Aboriginal or Torres Strait Islander (ATSI), Asian or South American <90cm; <80cm for females), or elevated waist circumference (≥ 94cm for males or if ATSI, Asian or South American ≥90cm; ≥80cm for females). HDL cholesterol were: normal HDL (≥1.0 mmol/L for males; ≥1.3 mmol/L for females), or low HDL (<1,0 mmol/l for males; <1.3 mmol/L for females). TG were: normal TG (<1.7 mmol/l), or hypertriglyceridaemia (≥1.7 mmol/l). BP were: normal BP (<130/85 mmHg and no anti-hypertensive medications), or high BP (≥130/85 mmHG or on anti-hypertensive medications) [40].

#### Statistical analysis

The statistical analysis was conducted in the following two stages:

Step 1: Descriptive statistics for HbA1c and FPG were obtained and normality was assessed for variables of interest (natural logarithm transformation was applied if variable was skewed). Differences between groups were then examined by Independent samples *t* test and χ^2^ test.

Step 2: Multiple logistic regression analyses were employed to obtain adjusted odds ratio (AOR) and 95% CI for the associations between serum 25OHD and having higher HbA1c and FPG, respectively. Three categories of variables were used in the regression models including:*socio-demographic variables* (age, sex, country of birth, IRSED, physical activity, smoking status, and season), *dietary factors* (dietary fiber, magnesium, alcohol, calcium, zinc, carbohydrate intake, energy intake, and under/over reporting of energy intake) and *biomedical variables* (MetS components: waist circumference (ethnic specific cut-offs), HDL cholesterol (normal/low), TG (normal/high), BP (normal/high), BMI and haemoglobin levels for the HbA1c model only). All these variables have a known association with FPG and HbA1c and have been previously reported in the literature.

The primary research variable of interest, serum 25OHD, was entered into the multiple regression model as 1) a continuous variable (10 nmol/L increments) and also as 2) a categorical variable based on 25OHD tertiles. For both continuous and categorical 25OHD, the multiple regression model was initially adjusted for the socio-demographic variables in Model 1; secondly both socio-demographic variables, dietary factors and haemoglobin levels (HbA1c model only) in Model 2; and finally all socio-demographic, dietary factors and biomedical variables altogether in Model 3. The IBM SPSS Statistics for Windows, Version 21.0, was used for the statistical analyses. The VHM survey employed the use of the multistage stratified cluster-sampling procedure. In order to adjust for the unequal selection probability due to this sampling method, complex samples analysis was used. Complex samples approach takes into account the complex survey sampling and selection probability used in the VHM survey. Variables (strata variable, weighting variable and clustering variable) describing the survey design in terms of stratification, clustering and multistage sampling were entered into the SPSS complex samples approach for generating sampling weights in estimation and standard errors. A two-tailed p value of less than 0.05 was accepted as statistical significance.

## Results

An overview of socio-demographic, dietary and clinical characteristics of subjects based on FPG and HbA1c levels are shown in Table 1 (please refer to S1 Table for the full socio-demographic, dietary and clinical characteristics of subjects). The prevalence of those with normal FPG (<5.6 mmol/L) was 84% and for high FPG (5.6–6.9 mmol/L) was 16%. 61% of the population had normal HbA1c (<5.6%), while 39% had high HbA1c (5.7–6.4%).

**Table 1:** Socio-demographic and clinical characteristics of participants by FPG and HbA1c.

	FPG (<5.6 mmol/L) n = 2866 (84%)	FPG (5.6–6.9 mmol/L) n = 527 (16%)	P value	HbA1c (<5.7%) n = 2068 (61%)	HbA1c (5.7–6.4%) n = 1325 (39%)	P value
	Mean (SE) *or* N (SE) %	Mean (SE) *or* N (SE) %		Mean (SE) *or* N (SE) %	Mean (SE) *or* N (SE) %	
Age (y)	42 (0.8)	52 (1.4)	<0.001	40 (0.9)	52 (0.9)	<0.001
BMI (kg/m^2^)	26.6 (0.2)	29.2 (0.4)	<0.001	26.2 (0.2)	28.8 (0.2)	<0.001
*Gender*			<0.001			0.494
Males	1285 (1.5) 81%	299 (1.5) 19%		1112 (2.5) 70%	472 (2.5) 30%	
Females	1663 (1.0) 91%	162 (1.0) 9%		1257 (2.4) 69%	577 (2.4) 31%	
*Country of birth*			0.031			<0.001
Born in Australia	2264 (0.7) 88%	320 (0.7) 12%		1868 (2.1) 72%	716 (2.1) 28%	
Born overseas	674 (1.9) 83%	134 (1.9) 17%		505 (3.6) 62%	303 (3.6) 38%	
*IRSED*			0.020			0.216
Most disadvantaged	705 (1.7) 83%	142 (1.7) 17%		545 (5.1) 64%	302 (5.1) 36%	
Disadvantaged	732 (1.0) 87%	108 (1.0) 13%		570 (3.8) 68%	270 (3.8) 32%	
Less disadvantaged	765 (1.3) 89%	98 (1.3) 11%		639 (4.1) 74%	224 (4.1) 26%	
Least disadvantaged	737 (1.2) 87%	106 (1.2) 13%		631 (3.3) 75%	212 (3.3) 25%	
*Smoking status*			<0.001			0.002
Current smoker	441 (2.2) 84%	86 (2.2) 16%		366 (3.7) 69%	161 (3.7) 31%	
Ex-smoker	733 (2.0) 80%	179 (2.0) 20%		575 (3.2) 63%	337 (3.2) 37%	
Non-smoker	1756 (0.9) 90%	195 (0.9) 10%		1418 (2.1) 73%	533 (2.1) 27%	
*25OHD concentration*						
Serum 25OHD (nmol/L)	56.7 (2.0)	52.1 (2.5)	0.081	57.2 (2.2)	53.6 (1.9)	0.208
25OHD tertiles			0.045			0.135
Low 25OHD (33 nmol/L)	933 (1.5) 84%	180 (1.5) 16%		745 (2.9) 67%	359 (2.9) 33%	
Medium 25OHD (54 nmol/L)	992 (1.3) 85%	168 (1.3) 15%		798 (3.5) 69%	32 (3.5) 31%	
High 25OHD (77 nmol/L)	1013 (1.5) 90%	116 (1.5) 10%		829 (2.4) 73%	300 (2.4) 27%	
*Dietary variable*						
Energy (kJ/d)	9687.4 (116.5)	9784.9 (164.9)	0.653	9904.4 (147.8)	9236.4 (145.6)	<0.001
*Biomedical factors*						
Waist circumference (cm)	88.0 (0.7)	96.9 (1.1)	<0.001	86.9 (0.7)	94.7 (0.9)	<0.001
Triglycerides (mmol/L)	1.2 (0.03)	1.5 (0.04)	<0.001	1.1 (0.03)	1.5 (0.04)	<0.001
HDL (mmol/L)	1.5 (0.02)	1.4 (0.03)	<0.001	1.5 (0.02)	1.4 (0.02)	<0.001
Systolic blood pressure (mmHg)	123 (0.6)	133 (1.1)	<0.001	123 (0.7)	128 (0.6)	<0.001
Diastolic blood pressure (mmHg)	73 (0.5)	77 (0.7)	<0.001	72 (0.5)	76 (0.5)	<0.001
Haemoglobin levels (g/L)	142.9 (0.4)	148.2 (1.1)	<0.001	144.2 (0.4)	142.4 (0.6)	<0.001

Data are presented as mean estimate (weighted) (%) for categorical variables, and mean estimate (weighted) and (SE) for normal continuous variables. Difference in the continuous and categorical variables between groups were assessed by independent samples t-test (natural logarithm transformation was used if the variable was not normal) and Chi-square test (association between FPG or HbA1c and categorical variables, with an emphasis on which category were more likely to have high FPG, or high HbA1c), respectively.

Legend: d, day; SE, standard error; min, minutes; wk, week.

### Association between serum 25OHD and FPG

When the serum 25OHD was entered as a continuous covariate to the multiple regression model, for every increment in serum 25OHD of 10 nmol/L, the odds of having higher FPG reduced by 9% (AOR 0.91, (0.86, 0.97); p = 0.002) after adjusting for socio-demographic variables, dietary factors, and biomedical variables in Model 3. For all models when serum 25OHD was entered as a categorical factor, compared with those people in the low 25OHD tertile, those with the high 25OHD tertile had a significantly reduced risk of higher FPG. More specifically, after adjustment for both socio-demographic, dietary factors and BMI in Model 2 the odds of having higher FPG reduced by 40% for those in high 25OHD tertile (AOR 0.60, (0.43, 0.83); p = 0.008) vs. low 25OHD tertile. After further adjustment for MetS components in Model 3, the AOR appeared relatively stable with a 39% reduced odds of higher FPG in the high 25OHD vs. low 25OHD tertile (AOR 0.61, (0.44, 0.84); p = 0.011) (Table 2).

**Table 2:** The association of serum 25OHD and FPG: Crude and adjusted odds ratio and their 95% CI based on logistic regression.

	**Model 1**		**Model 2**		**Model 3**	
	**Crude OR**	**95% CI**	**AOR**	**95% CI**	**AOR**	**95% CI**
**25OHD continuous** (10 nmol/L)	0.93	0.87, 1.01	0.91	0.86, 0.97	0.91	0.86, 0.97
P value	0.054		0.003		0.002	
	**Model 1**		**Model 2**		**Model 3**	
**25OHD tertiles**	**Crude OR**	**95% CI**	**AOR**	**95% CI**	**AOR**	**95% CI**
Low 25OHD (33 nmol/L) †	1.0		1.0		1.0	
Medium 25OHD (54 nmol/L) †	0.89	0.60, 1.31	0.87	0.57, 1.32	0.87	0.59, 1.29
High 25OHD (77 nmol/L) †	0.66*	0.46, 0.94	0.60*	0.43, 0.83	0.61*	0.44, 0.84
P value for trend	0.076		0.008		0.011	

Model 1: age, sex, country of birth, IRSED, physical activity, smoking status, season, BMI.

Model 2: Model 1 plus dietary fiber, magnesium, alcohol, calcium, zinc, carbohydrate intake, energy intake, under/over reporting of energy intake.

Model 3: Model 2 plus waist circumference, HDL cholesterol, TG, BP; all as categorical variables based on MetS cut-offs.

Legend: Crude OR, crude odds ratio; AOR, adjusted odds ratio; 1.0, lowest 25OHD served as the reference group.

Footnotes

*, significant in comparison to reference group at 5% significance level

†, median of the tertile group.

### Association between serum 25OHD and HbA1c

In Model 3 after adjustment for socio-demographic, dietary, and biomedical variables every 10 nmol/L increment in 25OHD also significantly reduced the odds of higher HbA1c by 6% (AOR 0.94, (0.90, 0.98); p = 0.009). In Model 2, after adjustment for socio-demographic variables, dietary factors, BMI and haemoglobin levels, there was a significantly reduced odds of having higher HbA1c by 33% in those with high vs. low 25OHD tertile. After further adjustment for MetS components in Model 3, a 26% reduced odds of higher HbA1c (AOR 0.74, (0.58, 0.93); p = 0.041) were found in the high 25OHD tertile group, compared to the low 25OHD tertile group (Table 3).

**Table 3:** The association of serum 25OHD and HbA1c: Crude and adjusted odds ratio and their 95% CI based on logistic regression.

	**Model 1**		**Model 2**		**Model 3**	
	**Crude OR**	**95% CI**	**AOR**	**95% CI**	**AOR**	**95% CI**
**25OHD continuous** (10 nmol/L)	0.93	0.88, 0.97	0.93	0.88, 0.97	0.94	0.90, 0.98
P value	0.002		0.002		0.009	
	**Model 1**		**Model 2**		**Model 3**	
**25OHD tertiles**	**Crude OR**	**95% CI**	**AOR**	**95% CI**	**AOR**	**95% CI**
Low 25OHD (33 nmol/L) †	1.0		1.0		1.0	
Medium 25OHD (54 nmol/L) †	0.78	0.56, 1.09	0.79	0.56, 1.11	0.83	0.58, 1.17
High 25OHD (77 nmol/L) †	0.68*	0.54, 0.86	0.67*	0.53, 0.85	0.74*	0.58, 0.93
P value for trend	0.007		0.005		0.041	

Model 1: age, sex, country of birth, IRSED, physical activity, smoking status, season, BMI.

Model 2: dietary fiber, magnesium, alcohol, calcium, zinc, carbohydrate intake, energy intake, under/over reporting of energy intake, haemoglobin levels.

Model 3: Model 2 plus waist circumference, HDL cholesterol, TG, BP; all as categorical variables based on MetS cut-offs.

Legend: Crude OR, crude odds ratio; AOR, adjusted odds ratio; 1.0, lowest 25OHD served as the reference group.

Footnotes

*, significant in comparison to reference group at 5% significance level

†, median for the tertile group.

## Discussion

The results from this population based study of adults from Victoria, Australia indicate that higher 25OHD levels were significantly related to a reduced risk of higher FPG and HbA1c levels. This significant inverse association persisted after the adjustment for a number of socio-demographic, dietary, and biomedical variables and MetS components. We found that those in the high 25OHD tertile had a 39% reduced risk of higher FPG and 26% reduced risk of higher HbA1c, when compared to the low 25OHD tertile. This was independent of MetS components, and haemoglobin levels in the HbA1c model, which have been found to confound the association between 25OHD and T2DM. Two recent meta-analyses [41–42] found no significant effect of vitamin D supplementation on IR [41], FPG [41, 42] or HbA1c [42]. Though, another found a beneficial effect of vitamin D supplementation on FPG and HbA1c, despite no effect on IR [43]. Our recent review summarised that although there were inverse associations between 25OHD and IR, the systematic reviews and meta-analysis in that review did not favour a casual role [44]. The present findings are in line with previous observational studies that found an inverse association between 25OHD status and high FPG levels [20, 45] and high HbA1c levels [46–51]. However, these studies adjusted for fewer variables, with smaller sample sizes than our study and were not population based [45, 49–51]. The differences in findings between both interventional and observational based studies indicates a need for good quality randomized controlled trials.

A number of inter-related factors contribute to the pathogenesis of T2DM, including the presence of MetS components. One prospective study found that those with IR and high FPG (5.6–6.9 mmol/L) had double the risk of worsened cardio-metabolic profile after nine years [52]. Obesity, dyslipidemia and hypertension are MetS components which are often found in those with pre-diabetes [53]. We adjusted for these variables in our analysis and found that the association between 25OHD and risk of T2DM still existed, irrespective of MetS. Other studies investigating 25OHD and risk of T2DM have adjusted for either none [47–49], or one MetS components [46, 54, 55], with one study accounting for all of the MetS components [8].

### Potential mechanisms

The beneficial effect of 25OHD in reducing risk of T2DM is likely due to its effect on insulin action. The expression of the vitamin D receptor (VDR) in pancreatic beta cells indicates the importance of vitamin D in beta cell function [56, 57] and insulin secretion [5]. During times of vitamin D deficiency, beta cell function is blunted and insulin secretion is diminished [58]. Vitamin D may also indirectly influence insulin action via a calcium mediated effect. Vitamin D tightly regulates calcium homeostasis, whereby intracellular calcium levels are required to ensure effective action of insulin within different tissues [56]. Vitamin D is also involved in the regulation of the renin angiotensin system [59], endothelial vasodilation and lipid levels [60] which are mechanisms relating to the MetS components. T2DM and MetS are inter-twined, wherein IR appears to be a key player in the development of both conditions [61, 62]. Though there are commonalties in the mechanisms underlying both conditions, our study found that even on adjustment for MetS components, the association between the high 25OHD tertile and lower odds of higher FPG and HbA1c levels persisted. This may potentially suggest that vitamin D has a beneficial role in T2DM, independent of MetS. However, the cross-sectional nature of this study does not provide further insight into this observation.

Low grade chronic inflammation is a hallmark of many chronic diseases and may precede T2DM [63] possibly via initiation of IR [64]. Vitamin D has a role in immunity, and cellular studies show consistent reduction of inflammatory markers following cholecalciferol supplementation [65]. Adequate circulating 25OHD levels are required to obtain optimal anti-inflammatory responses in the body [66], especially in those tissues like immune cells, where the enzymes for conversion of 25OHD to 1,25OH_2_D are present. However, the optimum 25OHD levels for modulating inflammation responses are yet to be determined [67]. There is plausible but not confirmatory evidence to suggest the value is around 75–80 nmol/L [68, 69]; a point where maximum suppression of parathyroid hormone is also expected [70]. In support, a recent review [71], and emerging randomized controlled trials [72, 73] found beneficial effects of vitamin D supplementation on IR and fat loss in those individuals who reached this value over the period of the trial [72, 73]. In the present study those in the highest 25OHD tertile had a median of 77 nmol/L, where reduced risk of higher FPG and HbA1c was observed.

### Limitations

The cross-sectional design does not afford causality of association, though we have controlled for several known confounders. The VHM did not collect information on supplement use, so we cannot separate the potential effects of vitamin D supplement and increased sun exposure on the higher 25OHD levels. Family history of T2DM may increase the risk of development of the disease [74], but unfortunately such information was not collected as part of the survey thus is a potential confounder. Approximately >75% of our sample were born in Australia, so potential effects of country of birth/ethnicity could not be determined.

Studies have indicated that 25OHD may vary due to the genetic variation of three polymorphisms in the vitamin D genes, including the vitamin D binding protein, VDR and the 25OHD activating enzyme [75, 76]. The concentration of the vitamin D binding protein may vary between individuals [77, 78] as well as fluctuations in its binding affinity [79]. There may also be a genetic association between VDR polymorphisms and T2DM. Recent evidence has found that the polymorphism of certain VDRs may increase susceptibility to T2DM [80] and certain MetS components [81]. Lastly, the conversion of 25OHD to its active form requires CYP27B1, an enzyme that may be affected by genetic variation [82]. These genetic variants were not studied as part of the VHM survey but certainly provide avenues for novel insights into the associations described here.

### Strengths

This study used a representative sample of adult Australians from one state of the country. We adjusted for a wide range of socio-demographic, biomedical and dietary factors. Our study appears to be one of the few [8] which has accounted for all MetS components when investigating associations between 25OHD and T2DM. We also adjusted for the possible misreporting of energy intakes [35]. That being said 24 h recalls in this study were obtained from a five-pass method which is considered the gold standard for dietary information. We also adjusted the HbA1c analysis for haemoglobin concentrations, since concomitant anaemia may be associated with inaccurate HbA1c levels [37, 38]. Other studies in the area have not adjusted for this factor [8, 46–49, 54, 55].

## Conclusions

Higher 25OHD status was associated with lower prevalent FPG as well as lower HbA1c concentrations after accounting for socio-demographic, lifestyle variables and MetS components. Such outcomes could suggest a direct role for the vitamin in the prevention of T2DM.

## Supporting information

S1 Table Socio-demographic and clinical characteristics of participants by FPG and HbA1c. Data are presented as mean estimate (weighted) (%) for categorical variables, and mean estimate (weighted) and (SE) for continuous variables. Difference in the continuous and categorical variables between groups were assessed by independent samples t-test and Chi-square test, respectively. Legend: d, day; SE, standard error; min, minutes; wk, week.
(DOCX)
