# Supplementary material for: Vitamin D status is inversely associated with markers of risk for type 2 diabetes: A population based study in Victoria, Australia
Source: PLoS One. 2017 Jun 2;12(6):e0178825. doi: 10.1371/journal.pone.0178825 (PMC5456387; doi:10.1371/journal.pone.0178825)
Supplement: S1 Table — Data are presented as mean estimate (weighted) (%) for categorical variables, and mean estimate (weighted) and (SE) for continuous variables. Difference in the continuous and categorical variables between groups were assessed by independent samples t-test and Chi-square test, respectively. Legend: d, day; SE, standard error; min, minutes; wk, week. (DOCX) [file pone.0178825.s001.docx]

|  | **FPG**  **(<5.6 mmol/L)**  n=2866 (84%) | **FPG**  **(5.6-6.9 mmol/L)**  n=527 (16%) | **P value** | **HbA1c**  **(<5.6%)**  n=2068 (61%) | **HbA1c**  **(5.7-6.4%)**  n=1325 (39%) | **P value** |
| --- | --- | --- | --- | --- | --- | --- |
|  | **Mean (SE) *or***  **N (SE) %** | **Mean (SE) *or***  **N (SE) %** |  | **Mean (SE) *or***  **N (SE) %** | **Mean (SE) *or***  **N (SE) %** |  |
| Age (y) | 42 (0.8) | 52 (1.4) | <0.001 | 40 (0.9) | 52 (0.9) | <0.001 |
| BMI (kg/m^2^) | 26.6 (0.2) | 29.2 (0.4) | <0.001 | 26.2 (0.2) | 28.8 (0.2) | <0.001 |
| *Gender* |  |  | <0.001 |  |  | 0.494 |
| Males | 1285 (1.5) 81% | 299 (1.5) 19% |  | 1112 (2.5) 70% | 472 (2.5) 30% |  |
| Females | 1663 (1.0) 91% | 162 (1.0) 9% |  | 1257 (2.4) 69% | 577 (2.4) 31% |  |
| *Country of birth* |  |  | 0.031 |  |  | <0.001 |
| Born in Australia | 2264 (0.7) 88% | 320 (0.7) 12% |  | 1868 (2.1) 72% | 716 (2.1) 28% |  |
| Born overseas | 674 (1.9) 83% | 134 (1.9) 17% |  | 505 (3.6) 62% | 303 (3.6) 38% |  |
| *IRSED* |  |  | 0.020 |  |  | 0.216 |
| Most disadvantaged | 705 (1.7) 83% | 142 (1.7) 17% |  | 545 (5.1) 64% | 302 (5.1) 36% |  |
| Disadvantaged | 732 (1.0) 87% | 108 (1.0) 13% |  | 570 (3.8) 68% | 270 (3.8) 32% |  |
| Less disadvantaged | 765 (1.3) 89% | 98 (1.3) 11% |  | 639 (4.1) 74% | 224 (4.1) 26% |  |
| Least disadvantaged | 737 (1.2) 87% | 106 (1.2) 13% |  | 631 (3.3) 75% | 212 (3.3) 25% |  |
| *Physical activity level* |  |  | 0.635 |  |  | <0.001 |
| Sufficient physical activity  (≥150 min/wk) | 1992 (1.0) 86% | 314 (1.0) 14% |  | 1660 (2.6) 72% | 646 (2.6) 28% |  |
| Insufficient physical activity  (<149 min/wk) | 763 (1.6) 88% | 108 (1.6) 12% |  | 590 (2.5) 68% | 281 (2.5) 32% |  |
| Inactive  (0 min/wk) | 180 (2.2) 85% | 31 (2.2) 15% |  | 106 (4.4) 50% | 106 (4.4) 50% |  |
| *Smoking status* |  |  | <0.001 |  |  | 0.002 |
| Current smoker | 441 (2.2) 84% | 86 (2.2) 16% |  | 366 (3.7) 69% | 161 (3.7) 31% |  |
| Ex-smoker | 733 (2.0) 80% | 179 (2.0) 20% |  | 575 (3.2) 63% | 337 (3.2) 37% |  |
| Non-smoker | 1756 (0.9) 90% | 195 (0.9) 10% |  | 1418 (2.1) 73% | 533 (2.1) 27% |  |
| *Season of biomedical examination* |  |  | 0.751 |  |  | 0.045 |
| Summer | 177 (3.2) 86% | 30 (3.2) 14% |  | 106 (7.8) 51% | 101 (7.8) 49% |  |
| Autumn | 706 (1.2) 88% | 99 (1.2) 12% |  | 552 (3.3) 69% | 253 (3.3) 31% |  |
| Winter | 983 (1.0) 86% | 160 (1.0) 14% |  | 852 (3.7) 74% | 291 (3.7) 26% |  |
| Spring | 1070 (1.5) 86% | 168 (1.5) 14% |  | 862 (3.2) 70% | 376 (3.2) 30% |  |
| *25OHD concentration* |  |  |  |  |  |  |
| Serum 25OHD (nmol/L) | 56.7 (2.0) | 52.1 (2.5) | 0.081 | 57.2 (2.2) | 53.6 (1.9) | 0.208 |
| 25OHD tertiles |  |  | 0.045 |  |  | 0.135 |
| Low 25OHD  (33 nmol/L) | 933 (1.5) 84% | 180 (1.5) 16% |  | 745 (2.9) 67% | 359 (2.9) 33% |  |
| Medium 25OHD  (54 nmol/L) | 992 (1.3) 85% | 168 (1.3) 15% |  | 798 (3.5) 69% | 32 (3.5) 31% |  |
| High 25OHD  (77 nmol/L) | 1013 (1.5) 90% | 116 (1.5) 10% |  | 829 (2.4) 73% | 300 (2.4) 27% |  |
|  |  |  |  |  |  |  |
| *Dietary variables* |  |  |  |  |  |  |
| Calcium (mg/d) | 917.1 (12.0) | 879.9 (22.1) | 0.011 | 924.6 (13.3) | 883.8 (15.8) | 0.009 |
| Magnesium (mg/d) | 414.9 (7.1) | 416.4 (9.2) | 0.850 | 422.8 (6.9) | 397.6 (6.6) | 0.001 |
| Dietary fiber (g/d) | 26.2 (0.4) | 26.8 (0.5) | 0.158 | 26.5 (0.4) | 25.8 (0.4) | 0.120 |
| Zinc (mg/d) | 13.4 (0.2) | 13.5 (0.4) | 0.266 | 13.6 (0.2) | 12.9 (0.3) | 0.001 |
| Carbohydrate (g/d) | 261.1 (3.3) | 253.7 (4.2) | 0.035 | 265.6 (4.2) | 248.0 (4.1) | <0.001 |
| Energy (kJ/d) | 9687.4 (116.5) | 9784.9 (164.9) | 0.653 | 9904.4 (147.8) | 9236.4 (145.6) | <0.001 |
| *Biomedical factors* |  |  |  |  |  |  |
| Waist circumference (cm) | 88.0 (0.7) | 96.9 (1.1) | <0.001 | 86.9 (0.7) | 94.7 (0.9) | <0.001 |
| Triglycerides (mmol/L) | 1.2 (0.03) | 1.5 (0.04) | <0.001 | 1.1 (0.03) | 1.5 (0.04) | <0.001 |
| HDL (mmol/L) | 1.5 (0.02) | 1.4 (0.03) | <0.001 | 1.5 (0.02) | 1.4 (0.02) | <0.001 |
| Systolic blood pressure (mmHg) | 123 (0.6) | 133 (1.1) | <0.001 | 123 (0.7) | 128 (0.6) | <0.001 |
| Diastolic blood pressure (mmHg) | 73 (0.5) | 77 (0.7) | <0.001 | 72 (0.5) | 76 (0.5) | <0.001 |
| Haemoglobin levels (g/L) | 142.9 (0.4) | 148.2 (1.1) | <0.001 | 144.2 (0.4) | 142.4 (0.6) | <0.001 |
